# Inflammatory Myoglandular Polyps: A Case Series of Four Patients and Review of the Literature

**DOI:** 10.1155/2010/984092

**Published:** 2010-04-11

**Authors:** Shoji Hirasaki, Hiromitsu Kanzaki, Minoru Matsubara, Seiyuu Suzuki

**Affiliations:** ^1^Division of Gastroenterology, Kubo Hospital, 1-1-19 Uchibori, Imabari 7992116, Japan; ^2^Second Department of Internal Medicine, Sumitomo Besshi Hospital, 3-1 Ohji-cho, Niihama 7928543, Japan

## Abstract

*Background*. Inflammatory myoglandular polyp (IMGP) is a nonneoplastic colorectal polyp. Only a small number of cases have been reported, and the pathogenesis remains unclear. *Methods*. We analyzed colonoscopy and histologic findings in 4 patients with IMGP. Histologic confirmation of the inflammatory granulation tissue in the lamina propria, proliferation of smooth muscle, and hyperplastic glands with variable cystic changes formed the criteria for the selection of patients. *Results*. We treated four cases of IMGP and reviewed the literature on this disease. Three cases were located in the sigmoid colon or descending colon. All 4 polyps were identified as red, pedunculated lesions. All 4 cases had no symptoms. In two cases, endoscopic findings of polyps were necessary to be differentiated from juvenile polyps. *Conclusions*. Pedunculated lesions are the main pattern of IMGP. An analysis of endoscopic and histologic features in IMGP of the colorectum revealed that colonic IMGPs resembled juvenile polyps. On colonoscopy, IMGP should generally be taken into consideration as a differential diagnosis of peduncular polyp.

## 1. Introduction

The first published report of inflammatory myoglandular polyp (IMGP) is attributed to S Nakamura. who reported on 32 patients with this disease [1]. Since then, there had been several case reports of IMGP treated with endoscopic resection or surgery [2–12]. IMGP is thought to be clinically and histologically benign. Therefore, endoscopic treatment should always be attempted in order to avoid surgery in these patients. We had once reported 2 patients with IMGP [11, 12]. We present a new case series of 4 patients with IMGP who underwent endoscopic polypectomy and investigate clinicopathologic and endoscopic findings.

## 2. Case Presentation

### 2.1. Case 1

**Figure 1:**
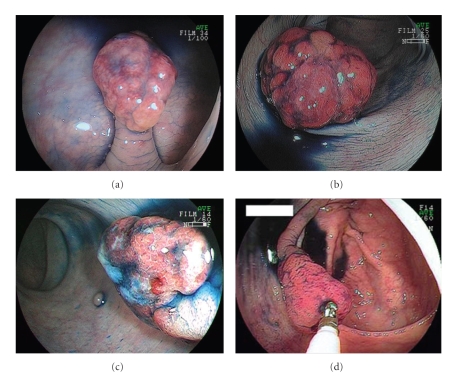
Endoscopic appearances of 4 inflammatory myoglandular polyps. All lesions were red and pedunculated polyps.

A 45-year-old woman visited our hospital for further evaluation of fecal occult blood in a yearly physical checkup. Colonoscopy revealed a red peduncular polyp, about 20 mm in diameter, in the sigmoid colon (Figure 1(a)). With conventional colonoscopy, the lesion did not show type III or IV pit pattern although magnifying colonoscopy was not performed. We speculated that this polyp was nonneoplastic. It was suspected to be an inflammatory polyp [IMGP or inflammatory fibroid polyp (IFP)] from endoscopic findings. Excluding the polyp, there was no lesion in the colorectum. Endoscopic polypectomy was performed. Histological examination of the specimen revealed inflammatory granulation tissue in the lamina propria, proliferation of smooth muscle, and hyperplastic glands with cystic change.

### 2.2. Case 2

**Figure 2:**
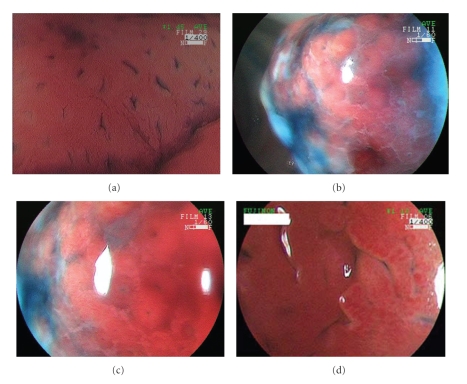
Magnifying colonoscopy appearance of three inflammatory myoglandular polyps. (a) In case 2, magnifying colonoscopy showed type I or II pit pattern. (b) In case 3, magnifying colonoscopy showed a red, slightly rugged surface consisting of an aggregation of smooth nodules. (c) Some normal round crypt openings were seen in the surface of the polyp by magnifying colonoscopy observation in case 3. (d) Magnifying colonoscopy showed a red, slightly rugged surface consisting of an aggregation of smooth nodules in case 4.

A 50-year-old man visited our hospital for further evaluation of fecal occult blood in a yearly physical checkup. Colonoscopy revealed a red, hard peduncular polyp, about 20 mm in diameter, in the descending colon (Figure 1(b)). Magnifying observation (EC-450ZH, Fujinon Toshiba ES Systems) showed type I or II pit pattern (Figure 2(a)). We speculated that this polyp was nonneoplastic. It was suspected to be an inflammatory polyp from endoscopic findings although it should be distinguished from a juvenile polyp. Endoscopic polypectomy was performed. Histological examination of the specimen revealed inflammatory granulation tissue in the lamina propria, proliferation of smooth muscle, and hyperplastic glands with cystic change (Figures 4(a) and 4(b)). 

### 2.3. Case 3

**Figure 3:**
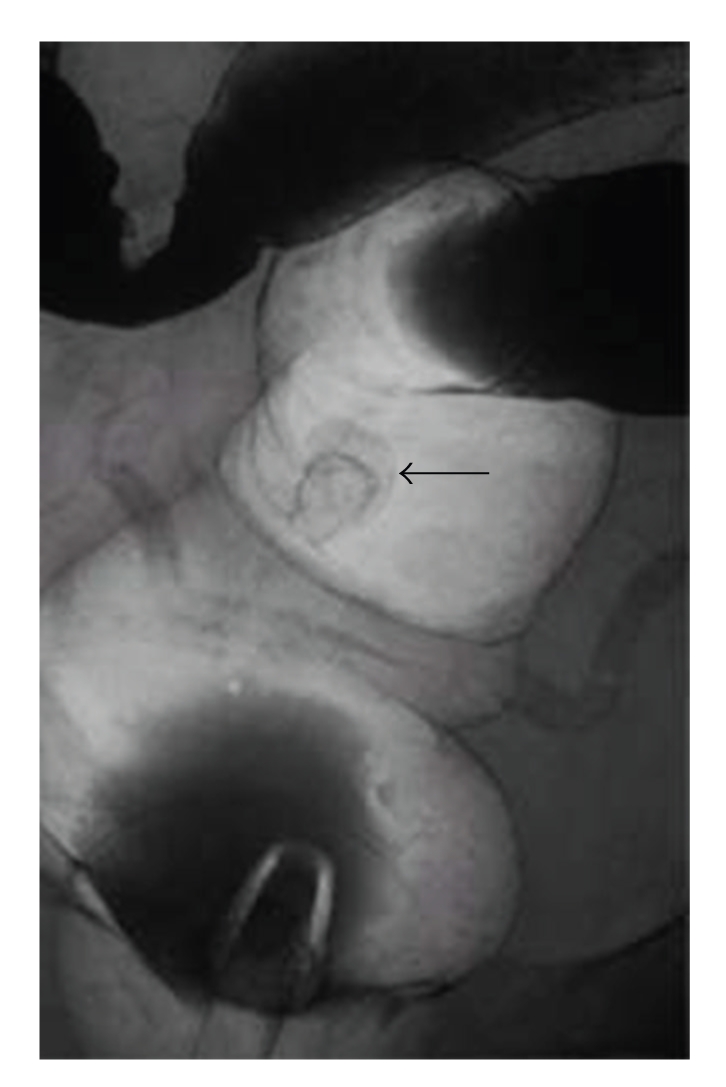
In case 3, an air contrast barium enema also revealed a pedunculated polyp like a snowman shape (arrow) in the sigmoid colon.

**Figure 4:**
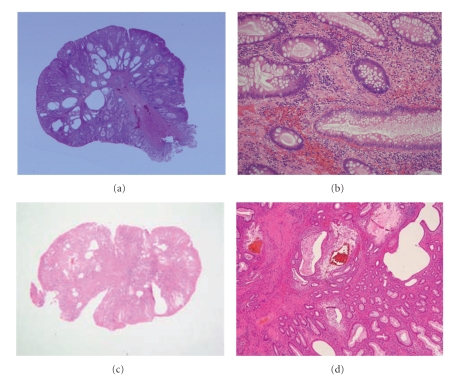
In case 2, histological examination of the specimen revealed hyperplastic glands with variable cystic changes (a), inflammatory granulation tissue in the lamina propria, proliferation of smooth muscle (b). In case 3, (c) hyperplastic glands with variable cystic changes were seen in the superficial portion in the polyp. Inflammatory granulation tissue in the lamina propria and proliferation of smooth muscle were also seen in the resected specimen (d).

A 49-year-old man consulted a physician for further evaluation of fecal occult blood in a yearly physical checkup. Colonoscopy revealed a red, hard, spherical peduncular polyp with erosion and mucous exudation, about 20 mm in diameter, in the sigmoid colon (Figure 1(c)). Magnifying observation (EC-450ZH, Fujinon Toshiba ES Systems) revealed a red, slightly rugged surface consisting of an aggregation of smooth nodules (Figure 2(b)) with some normal round crypt openings (Figure 2(c)). We speculated that this polyp was nonneoplastic. It was suspected to be an inflammatory polyp from endoscopic findings although it should be distinguished from a juvenile polyp. An air contrast barium enema also revealed a pedunculated polyp like a snowman shape in the sigmoid colon (Figure 3). Endoscopic polypectomy was performed. Histological examination of the specimen revealed inflammatory granulation tissue in the lamina propria, proliferation of smooth muscle, and hyperplastic glands with cystic change (Figures 4(c) and 4(d)).

### 2.4. Case 4

A 78-year-old man consulted a physician for further evaluation of fecal occult blood in a yearly physical checkup. Colonoscopy revealed a red, hard, conic peduncular polyp, about 20 mm in diameter, in the ascending colon (Figure 1(d)). With conventional colonoscopy, the lesion did not show type III or IV pit pattern. Magnifying observation (EC-450ZH, Fujinon Toshiba ES Systems) revealed a red, slightly rugged surface consisting of an aggregation of smooth nodules (Figure 2(d)). We speculated that this polyp was nonneoplastic. It was suspected to be an inflammatory polyp (IMGP or IFP) from endoscopic findings. Excluding the polyp, there was no lesion in the colorectum. Endoscopic polypectomy was performed. Histological examination of the specimen revealed inflammatory granulation tissue in the lamina propria, proliferation of smooth muscle, and hyperplastic glands with cystic change.

## 3. Management

All four patients underwent endoscopic polypectomy. There were no complications. The postpolypectomy courses were uneventful. There are no reports of IMGP recurrence after endoscopic resection, but there are no reports that describe the result of long-term follow-up study. Follow-up colonoscopy was commenced. The mean follow-up period was 1280 days (range 2–5 years); there were no local recurrences or new IMGP appearance after polypectomy in all four patients.

## 4. Discussion

IMGP is a nonneoplastic colorectal polyp, first described by Nakamura et al. [1]. IMGP is solitary, pedunculated and rarely, covered by a fibrin cap, and follows a benign course. Also, IMGP has no association with inflammatory bowel diseases and is located not only in the rectosigmoid, but also in the descending and transverse colon [3]. In the present cases, 2 cases were located in the sigmoid colon; one case was located in the descending colon and another one located in the ascending colon. Although the pathogenesis of IMGP remains unknown, Nakamura [1] proposed that chronic trauma from intestinal peristalsis may contribute to the pathogenesis of IMGP. IMGP is present mostly in middle age, predominantly in men [3]; however, cases have been described from young age to even 78 years of age. IMGPs have a broad range of sizes (0.4–2.5 cm) [1, 4–6]. Kayhan et al. [7] have reported a case of large IMGP (>6 cm) that was too large to be removed endoscopically and was thus treated with surgical resection. The most common location is the sigmoid colon and IMGPs of the large intestine are predominantly in the distal colon. 

### 4.1. Diagnosis

In most cases, the presenting signs and symptoms of IMGP are nonspecific. In a recent systematic review, Fujino et al. [8] described the most common symptoms of patients with IMGP as positive fecal occult blood (29%), and hematochezia (23%); less commonly, patients present with abdominal pain, constipation and anemia [2]. Small IMGPs in the colon are usually asymptomatic and often detected incidentally on barium enema or endoscopy [8–10]. Endoscopic characteristic findings include: (1) pedunculated or semipedunculated, (2) red, and (3) smooth, spherical, and hyperemic surface with patchy mucous exudation and erosion [8, 9]. It is difficult to distinguish IMGPs from juvenile polyps [13, 14] or inflammatory fibroid polyps [15, 16] according to endoscopic findings (Table 1). It is necessary to distinguish IMGPs from juvenile polyps because juvenile polyps sometimes recur after endoscopic resection [17] and recently juvenile polyps are suspected that they have neoplastic potential [18]. 

Moriyama et al. reported magnifying endoscopic findings of 5 IMGPs in 2003 [9]. They described that magnifying observation revealed a slightly rugged surface consisting of aggregated smooth nodules with enlarged round or oval crypt openings. In case 3 and case 4, magnifying endoscopic findings were the same as Moriyama's description. However, in case 2, enlarged round or oval crypt openings were found; however, aggregated smooth nodules were not seen. We had once reported a unique lobulated IMGP that had a red, slightly rugged surface component without normal mucosal structure and smooth white nodules with enlarged round or oval crypt openings [11]. There have been few reports on magnifying endoscopic findings of IMGP. To clarify the characteristic magnifying endoscopic findings of IMGP, we should accumulate and analyze many cases of IMGP. 

IMGP is characterized by inflammatory granulation tissue in the lamina propria, proliferation of smooth muscle, and hyperplastic glands with variable cystic changes. Histological findings of IMGP are similar to those of juvenile polyp [14]. The diagnosis of colorectal IMGPs could seldom be made by endoscopic biopsy and the final diagnosis of colonic IMGP depends on the pathological findings of EMR or endoscopic polypectomy specimens because the biopsy is often not deep enough to obtain the tissue showing proliferation of smooth muscle or hyperplastic glands with variable cystic changes. 

There have been few reports on barium enema characteristic findings of IMGP. We had once reported radiographic findings of 2 IMGP cases [11, 12]. An about 40 mm in diameter, pedunculated, lobulated polyp located in the ascending colon in one case [11], and an about 20 mm in diameter, spherical peduncular polyp located in the descending colon in another case [12]. We showed four new IMGP cases in this literature, and presented a radiographic finding of an about 20 mm in diameter, pedunculated polyp like a snowman shape, located in the sigmoid colon in case 3.

### 4.2. Treatment

IMGP of the large intestine can best be removed endoscopically, because it is thought to be clinically and histologically benign. IMGP has never been reported to accompany neoplasia to date [7]. Most Japanese cases have been treated with polypectomy or endoscopic mucosal resection (EMR) [5, 7–12].

## 5. Conclusions

In conclusion, an analysis of endoscopic and histologic features in IMGP of the colorectum revealed that colonic IMGPs resembled juvenile polyps. IMGP should generally be taken into consideration as a differential diagnosis of peduncular polyp of the colon. 

## Figures and Tables

**Table 1:** Characteristics of inflammatory myoglandular polyp, juvenile polyp, and inflammatory fibroid polyp.

Polyp [references]	Color	Shape	Common site	Incidence of hematochezia	Incidence of blood in the stool	Histological findings
IMGP [8]	Red	Ip	Distal Colon	23%	29%	Inflammatory granulation
						Proliferation of smooth muscle
						Hyperplastic glands with cystic changes

Juvenile Polyp [13, 14]	Red	Ip	Distal Colon	100%	Not described	Cystic architecture
						Mucus-filled glands
						Infiltration of inflammatory cells

IFP [15, 16]	Red or covered with normal mucosa	Ip	Proximal Colon	14%	14%	Proliferation of fibroblasts
						Infiltration of inflammatory cells (plasma cells and eosinophils)

IMGP: inflammatory myoglandular polyp.

IFP: inflammatory fibroid polyp.
